# Supplementary material for: Are carbon emissions trading and green financial instruments synergistic? -Comprehensive quantitative research based on content analysis
Source: PLoS One. 2024 Mar 7;19(3):e0298601. doi: 10.1371/journal.pone.0298601 (PMC10919601; doi:10.1371/journal.pone.0298601)
Supplement: S1 Data — (ZIP) [file pone.0298601.s001.zip › Data/地级市Z_F工作报告.zip!/║ú─╧/╚2╤╟╩╨/└┤╫╘╣1⁄2╓┌║┼í╛┬φ┐╦╩2╛▌═°í┐_2019.docx]

各位代表：

现在，我代表三亚市人民政府，向大会报告工作，请予审议，并请市政协委员和其他列席人员提出意见。

一、2018年工作回顾

2018年是贯彻党的十九大精神的开局之年，也是我国改革开放40周年和海南建省办经济特区30周年。一年来，在党中央、国务院和省委省政府、中共三亚市委的坚强领导下，在市人大、市政协的监督支持下，我们以习近平新时代中国特色社会主义思想为指导，全面贯彻党的十九大和十九届二中、三中全会精神，深入学习贯彻习近平总书记“4·13”重要讲话、中央12号文件精神，认真落实《中国（海南）自由贸易试验区总体方案》及中央有关部委和省委省政府关于深化改革开放的政策文件，抓紧推进涉及三亚的重点工作和先导性项目，力争在海南自由贸易试验区和中国特色自由贸易港建设中当好标杆。初步核算，2018年全市实现生产总值595.5亿元，增长7.2%，三次产业结构为11.5:19.8:68.7；地方一般公共预算收入100.4亿元，增长13.3%，首次突破百亿元大关；社会消费品零售总额241.8亿元，增长8%；城镇新增就业岗位3.5万个，城镇登记失业率1.47%；城镇常住居民人均可支配收入36417元，增长8.3%；农村常住居民人均可支配收入15773元，增长8.3%；居民消费价格涨幅为2.5%。

（一）自贸试验区建设开启新征程

先导性项目开局良好。成功举办首届海南岛国际电影节。引入市场化运营机制，与招商局集团、中化集团成立合资平台公司，负责南繁科技城、深海科技城、大学城和全球动植物种质资源引进中转基地开发建设，完成总体规划、控制性详细规划和产业规划编制工作，完成三亚市港口规划（包括南山港）编制工作。通过国际征集方式，高水平编制完成三亚总部经济及中央商务启动区城市设计暨概念性建筑方案、控制性详细规划。民航三亚区域管制中心接管湛江区域的高空空域，三亚进出港航路更加顺畅。

招商引才成效显著。积极开展“百日大招商”活动，成立招商引资工作机构，与中船重工、中船工业、中粮集团、平安集团、太平集团、中信集团、保利国际、光大集团、腾讯公司、复星集团及英国默林、托马斯库克、伊诺华、香港富德、富华集团等88家企业会见洽谈，其中世界500强21家、中国500强30家，洽谈合作项目276项，签署战略合作协议45家，14家总部企业注册落地，其中8家已导入产值。太平金融、阳光人寿、瑞泽新型建材、新道科技、京东佳禹、中交海投、中船重工物贸、东华博育云等8家企业被省政府首批认定为总部企业，其中太平金融被认定为跨国公司地区总部。分两批集中开工自贸试验区建设项目50个，总投资约176亿元。落实“百万人才进海南”行动计划，出台引才政策措施，优化人才发展环境，全市共办理人才落户10172人，办理外国人居留许可2141人次。

法治化、国际化、便利化营商环境正在形成。以入选全国第二批营商环境试评价城市为契机，深化“放管服”改革，出台《三亚市优化审批服务20条》，有序开展商事制度改革、审批服务事项“一窗受理”“不见面审批”“一网通办”等工作，在全省率先推行“一枚共享章”制度，在全国首创24小时12345行政审批专席受理代办制，企业注册登记、项目审批效率得到提升，全年新增企业9480家，增长42%。积极稳妥推进国税地税征管体制改革，国税地税机构顺利合并。组建三亚市国际商事纠纷调解中心、海南国际仲裁院三亚分院，商事纠纷解决机制正逐步完善。完成了国务院关于口岸通关时间再压缩三分之一的任务，排名全省前列。落实减税降费政策，支持民营经济发展，去年累计减免税费52.7亿元。拓宽融资途径，海南股权交易中心三亚分中心正式成立，企业在资本市场直接融资6.9亿元。

对外开放明显加快。全年实际利用外资1亿美元，增长2.5倍；实现外贸进出口总值62.9亿元，增长25.8%。凤凰机场年旅客吞吐量突破2000万人次，三亚成为全国首个空港年吞吐量突破2000万的非省会地级城市。新开通至英国伦敦等13条境外航线，全年运行境外航线达36条，被列为空港型国家物流枢纽承载城市。海南首家中外合资旅行社——酷怡国际旅行社（三亚）有限公司已正式开业。享受59国免签新政来我市旅游的外国人超过20万人次，全市接待入境游客81.7万人次，再创历史新高。亚洲彩虹盛典在国内首次举行，成功举办三亚▪财经国际论坛、世界小姐总决赛、克利伯环球帆船赛、世界大学生电竞联赛全球总决赛等国际性活动和两届三亚国际音乐节。三亚市中医院阿拉木图中医中心建成开诊，医疗健康服务走出国门。

（二）产业转型发展呈现新局面

主动推进房地产业调整。坚决贯彻落实房地产限购政策，狠抓房地产市场调控、监管整治和结构调整，2018年全市新建商品房网签销售面积下降49.5%，房地产业提供的税收收入在全市税收中所占比重逐步下降。除房地产投资以外的其他投资同比增长12%，超过全年投资预期目标。

旅游业提质升级。全年接待国内外游客2242.6万人次（含一日游人数），实现旅游总收入514.7亿元，同比分别增长11.3%和17%。邮轮、游艇、婚庆、亲子旅游等业态快速发展，亚特兰蒂斯项目正式运营，海昌梦幻海洋不夜城开始试运营，亚龙湾被评为海南唯一的国家级旅游度假区。离岛免税购物政策进一步放宽，全年免税销售额增长32.1%。积极推动“大三亚”经济圈建设，加强旅游市场联合宣传推介，开展旅游联合检查执法，开通三亚至邻县主要旅游景点的公交线路。三亚放心游消费监管系统覆盖全市12家海鲜广场、227家海鲜排档，实现消费数据联网实时监督和价格飘红预警，真正管好“一条鱼半斤虾”。开通三亚旅游诚信管理系统，持续开展旅游暗访，加强涉旅舆情监测，加大联合执法力度，及时高效处理投诉，旅游市场秩序总体保持稳定。

现代服务业加快发展。会展业规模持续壮大，全年举办会展活动8500余场，包括千人以上会议70场、200个标准展位展览40场，其中中国（三亚）国际大健康与美容产业博览会成为全省首个通过UFI认证的品牌博览会。金融业持续发展，亚太金融（基金）小镇入驻企业93家，创新推出游艇关税保函等业务，加强对小微企业的贷款支持，积极服务实体经济，全年金融业增加值63.9亿元，占全市GDP的10.7%。医疗健康产业加快发展，市中医院通过国家中医药服务贸易先行先试骨干机构验收，中医药健康旅游示范区加快建设。

高新技术产业渐显成效。新增3家高新技术企业，新成立5家高端科研机构，引进了中科院种子创新研究院（筹）和遗传与发育生物学研究所。中船重工深远海试验场（水声一期）和中船重工海山科技园项目开工建设。中科院深海所“深海勇士”号载人潜水器完成第100次下潜，标志着我国载人深潜装备的研发水平向前迈进一大步。互联网“双创”中心投入使用，启迪之星、京东旅游生态产业基地等孵化器入驻率均超过80%，互联网信息产业园入园企业产值超过50亿元，比上年增长4倍以上。海南信息安全基地建成开园，40余家网信企业签约入驻。完成海南卫星星座规划和总体设计，遥感大数据云服务平台上线试运行。

（三）城乡融合发展形成新格局

“五网”基础设施建设扎实推进。有轨电车示范线实现试运行。南山“四个一”交通配套工程、海榆东线海棠湾至亚龙湾段道路改造、海榆东线亚龙湾至吉阳转盘道路改造等工程实现通车，亚龙湾第二通道等项目建设有序推进。公共交通在全省实现“九个率先”，全市行政村公交车覆盖率达100%，微公交、网约公交为市民群众提供了便利出行方式。加快推进城乡供水一体化，福海苑水质净化厂扩建等工程建设完成，荔枝沟水质净化二厂建设加快推进，北部山区饮水安全工程实现功能性通水，农村供水自来水普及率达88.45%，255个自然村实现“同网同质同价同服务”。电网建设扎实推进，全年户均停电时间小于12小时，中心城区户均停电时间小于3小时。城区小区和村委会及周边区域高速光纤和4G信号覆盖率达100%。

大力实施乡村振兴战略。出台《实施乡村振兴战略行动方案》《乡村振兴战略规划（2018-2022）》。全市农林牧渔业总产值105.7亿元，增长4.6%。积极推进“果农+互联网+国际物流”电商平台建设，助推三亚热带水果抢占市场。加强村庄规划建设管理，引入大企业带动，扎实推进34个美丽乡村建设，西岛社区、博后村入选海南省五星级美丽乡村。海棠湾水稻国家公园获批海南首家农旅融合国家4A级旅游景区，中廖村民宿获得海南省第一批民宿创建示范点称号，全年乡村旅游接待游客361万人次。

（四）三大攻坚战取得新成效

打好防范化解重大风险攻坚战。加强政府债务管理，规范融资举债行为，积极争取上级财力支持和债券资金，加快化解存量债务。坚决打击房地产投机炒作，加强金融监管和风险防范统筹协调，依法稳妥推进历史遗留问题处置。

打好精准脱贫攻坚战。成立全市打赢脱贫攻坚战指挥部，选派驻村工作队、帮扶责任人下沉一线驻村扶贫。在全省率先建成扶贫大数据系统，深入开展“漏评”“错退”排查工作，新识别贫困人口67户227人。积极发挥企业帮扶作用，着力推进就业扶贫、产业扶贫、旅游扶贫、消费扶贫，严格落实教育、医疗、住房安全“三保障”政策。在全省率先开展“社工扶贫”和开通扶贫公交，实施“先种养后奖补”产业扶贫模式。截至去年底，全市贫困发生率从2014年的2.11%降至0.09%。

打好污染防治攻坚战。坚决落实中央环保督察和国家海洋督察整改任务，督察移交信访件已100%办结，中央环保督察93项整改措施中已有54项落实到位，国家海洋督察38项整改措施中已有22项落实到位，4项基本落实到位。全市共拆除违建72.9万平方米，实现新增违建零增长目标。全面落实秸秆、垃圾禁烧和烟花爆竹禁放工作，强化对建筑工地等方面的监管，全市PM2.5年均浓度为15微克/立方米，环境空气质量优良天数比例达到98.1%。加快推进内河水环境治理，实施污水截流整治、水系连通、河流生态修复等工程，开展集中式饮用水水源地环境保护专项行动，基本建成城区污水主管网，启动实施全域范围的农村污水处理工程，地表水优良率91.7%，近岸海域水质优良率92.9%。建成全市“村收集、区转运、市处理”的垃圾收运体系，生活垃圾无害化处理率达到100%。全年完成造林绿化1400余亩，花卉种植8200余亩，完成青梅港红树林湿地保护工程，对严重影响景观的22个山体实施了修复。在中国生态文明论坛年会上，三亚被评为“2018美丽山水城市”。

（五）民生福祉谱写新篇章

加大民生投入力度。2018年全市民生支出139.6亿元，增长39.4%，占全市地方一般公共预算支出的78.1%，其中投入50.8亿元落实十大为民办实事事项，重点支持教育、医疗卫生、社会保障、住房保障、脱贫攻坚、交通等民生事业发展。

着力完善公共服务与社会保障。新增公办幼儿园5所，市第六中学与西南大学、市实验中学与海南中学合作办学顺利开展，人大附中三亚学校、上海外国语大学三亚附中等项目加快推进。三亚市正式接管省第三人民医院，上海儿童医学中心托管的三亚市妇女儿童医院新院区建成开诊，市疾病预防控制中心新院区开工建设，基层医疗卫生机构标准化建设加快推进。以医保支付方式改革为抓手，推进紧密型医联体建设，获国务院通报表扬。加快推进凤凰海岸、月川、南边海环河口等棚户区改造建设，完成棚改任务6519户，在建安置区项目13个。开工新建各类保障房10950套，改造农村危房198户。市政府保基本蔬菜平价网点已达到74个，菜价保持总体稳定。社保体系日益完善，参保覆盖面进一步扩大，养老金标准进一步提高。大力支持社会救助体系建设和慈善事业发展，妇女儿童、老龄等事业取得新成绩。鼓励和引导青少年在创新创业、创文巩卫、脱贫攻坚等方面发挥积极作用，志愿服务成为三亚的一道靓丽风景。

社会治理进一步强化。加强大数据应用，三亚人口动态监测系统投入试运行。“多村（居）一中心”城乡社区服务试点工作有序开展。积极开展信访矛盾化解攻坚战，打造“网上信访”主渠道，落实信访工作责任，解决了一批群众关心的现实问题。初步形成较为完善的社会组织体系，在社会治理方面发挥了积极作用。推进综治中心、雪亮工程、网格化管理“三位一体”新机制建设，完善“路长制”，推动警力下沉、警务前移，强化社会治安综合治理。深入开展扫黑除恶专项斗争，持续开展禁毒三年大会战，严厉打击各类违法犯罪活动。在全省率先完成城市安全风险评估，安全生产形势保持总体稳定。大力开展国防教育、国防动员、人民防空、民兵预备役、双拥共建等工作，民族宗教、外事侨务、防灾减灾、食药安全、气象、统计、档案、史志等工作取得新进步。

（六）政府自身建设迈出新步伐

加快建设法治政府。认真执行市人大及其常委会的决议、决定，自觉接受市人大、市政协的监督，全年办复市人大代表建议119件、政协提案174件，办复率100%，满意和基本满意率达到98%以上。根据《三亚市法治政府建设实施方案》明确的115项任务，成立领导小组，抓紧推动落实。出台了《三亚市政府规章制定程序规定》，配合市人大制定烟花爆竹燃放安全管理、公园管理、餐厨废弃物管理等方面的地方性法规。市、区、基层公共法律服务体系不断完善，法律服务水平进一步提升。深入推进综合行政执法体制改革，进一步完善依法行政考核评价体系，全面提升政府机关依法行政、规范执法的能力和水平。

打造廉洁高效政府。按照省委、省政府和市委的部署，加快推进政府机构改革。着力提升政府执行力，强化督查督办，2018年政府工作报告分解的222项任务已基本完成。持之以恒加强作风建设，加强政府信息公开、政府采购管理和投资评审管理，规范公共资源交易管理，提升财政资金使用效益。严肃查处各类违法违纪案件，解决了一批损害群众利益的突出问题。

各位代表，过去一年的成绩，是习近平总书记“4·13”重要讲话、中央12号文件和《中国（海南）自由贸易试验区总体方案》在三亚落地实践的结果，是省委省政府、中共三亚市委坚强领导的结果，是全市人民用勤劳、智慧、勇气干出来的！在这里，我谨代表市政府，向全市各族人民，向人大代表、政协委员，向各民主党派、工商联、无党派人士，向各人民团体和社会各界人士，向全市公安政法干警、消防救援指战员，向中央和省驻三亚各单位，向驻市解放军和武警部队官兵，向长期关心支持三亚发展的港澳同胞、台湾同胞、海外侨胞及国际友人，表示衷心的感谢！

同时，我们也清醒认识到，三亚在发展中还存在不少矛盾和问题，在此基础上建设自贸试验区难度不小。一是房地产业转型调整造成的缺口较大，旅游业、现代服务业、高新技术产业等发展壮大仍需时日，短期内难以完全替代，经济下行压力还比较大，同时仍有一些历史遗留问题需妥善处置。二是旅游发展硬件、软件均存在短板，具有国际水准的高端消费项目较少，旅游消费亟待升级。三是经济外向度低，进出口贸易规模小、层次低，利用外资水平不高。四是营商环境亟待优化，政府的执行力和行政效能、干部的知识结构和能力水平需要提升，符合国际标准的法律、财会、咨询等专业服务基础薄弱，高端专业人才缺乏，与自贸试验区建设需求差距很大。五是教育、医疗等社会事业发展滞后，公共服务还存在明显短板，社会治理管理和社会文明水平有待提升。六是生态环保工作还存在薄弱环节，局部环境质量不容乐观。对此，我们将高度重视，认真解决。

二、2019年工作总体要求

2019年是新中国成立70周年，是海南推进全面深化改革开放“政策落实年”，也是高标准高质量建设自由贸易试验区、探索建设中国特色自由贸易港的关键一年。做好今年的工作，关系长远，意义重大。今年政府工作的总体要求是：坚持以习近平新时代中国特色社会主义思想为指导，全面贯彻落实党的十九大和十九届二中、三中全会精神，深入学习贯彻习近平总书记“4·13”重要讲话、中央12号文件、《中国（海南）自由贸易试验区总体方案》等政策文件，落实省委七届历次全会和市委七届历次全会的部署，统筹推进“五位一体”总体布局，协调推进“四个全面”战略布局，抓住自贸试验区建设的机遇，按照高质量发展的要求，大胆探索创新，围绕“一个核心、两个环境、三个产业、四个平台、五个基础”，统筹推进稳增长、促改革、调结构、惠民生、防风险工作，进一步稳就业、稳金融、稳外贸、稳外资、稳投资、稳预期，加快推进世界级滨海旅游城市建设，力争在全面深化改革开放、建设现代化经济体系、推动人与自然和谐发展、满足人民美好生活需要等方面走在全省前列，在“大三亚”经济圈中发挥牵头示范作用，在海南自由贸易试验区和中国特色自由贸易港建设中打造标杆，在践行“三区一中心”定位中当好先锋，在争创新时代中国特色社会主义生动范例中勇做“排头兵”，成为海南打造展示中国风范、中国气派、中国形象的靓丽名片的引领者。在工作中，我们要把握好以下六点：

（一）听党指挥，坚决贯彻落实习总书记和中央指示精神

增强“四个意识”，坚定“四个自信”，自觉维护党中央权威和集中统一领导，始终在思想上政治上行动上同以习近平同志为核心的党中央保持高度一致。将学习贯彻习近平总书记“4·13”重要讲话和中央12号文件精神，与学习贯彻习近平新时代中国特色社会主义思想、学习贯彻党的十九大和十九届二中、三中全会精神结合起来，在“学懂弄通做实”上下功夫，在其指引下推进三亚全面深化改革开放。

（二）敢闯敢试，积极推进制度创新

习总书记在“4·13”重要讲话中指出，海南全岛建设自由贸易试验区，要以制度创新为核心。我们必须进一步解放思想，发扬敢闯敢试、敢为人先、埋头苦干的特区精神，围绕自贸试验区建设的重点领域、关键环节，结合三亚实际和发展需求，学习借鉴国内外先进经验，积极争取开展先行先试、制度创新，在守好安全底线的前提下，着力推动人员、货物、资金、信息等四个要素自由流动、自由流通，争当新时代改革开放的开拓者。

（三）补齐短板，打造一流营商环境

习总书记在“4·13”重要讲话中指出，海南要加快形成法治化、国际化、便利化的营商环境和公平开放统一高效的市场环境。与自贸试验区建设的需求相比，三亚发展基础薄弱，土地资源、生产资源和市场主体、人才、技术、资本等要素短缺，必须把优化营商环境作为发展的“生命线”来抓。要对标国际一流标准，加快补齐短板，充分发挥市场在资源配置中的决定性作用，降低企业交易成本，提升交易效率，努力打造具有国际竞争力的营商环境。

（四）持之以恒，不断优化产业结构

习总书记在“4·13”重要讲话中指出，海南要坚决贯彻新发展理念，建设现代化经济体系，在推动经济高质量发展方面走在全国前列。去年以来，我市按照中央要求和省里的部署，坚决转方式、调结构，实施房地产业调控和产业结构调整，取得了初步成效。接下来，一定要坚持顶住转型阶段的压力和阵痛，抓住自贸试验区建设的历史机遇，逐步摆脱对房地产业的依赖，加快建立开放型经济新体制，释放开放创新的活力，实现新旧动能转换，推动经济高质量发展。

（五）坚守红线，加强生态文明建设

习总书记在“4·13”重要讲话中指出，海南要牢固树立和全面践行绿水青山就是金山银山的理念，在生态文明体制改革上先行一步，为全国生态文明建设作出表率。优越的生态环境是三亚发展最大的资本和优势，一定要以习近平生态文明思想为指引，坚定不移走生产发展、生活富裕、生态良好的文明发展道路，将生态文明理念贯穿到参与自贸试验区建设的全过程，积极探索生态绿色发展的新模式。

（六）以人为本，持续改善人民生活

习总书记在“4·13”重要讲话中指出，海南要坚持以人民为中心的发展思想，不断满足人民日益增长的美好生活需要。我们要时刻牢记党的宗旨和发展的根本目的，努力补齐民生短板，不断增进民生福祉，让人民群众更直接、更深切地感受到改革开放和自贸试验区建设带来的成果，赢得更多的支持和参与，形成共建共享的良好局面。

三、2019年重点工作任务

2019年三亚发展的主要预期目标是：地区生产总值增长10%，固定资产投资增长10%，社会消费品零售总额增长10%，地方一般公共预算收入增长8.5%，货物和服务进出口总额增长15%，外商直接投资翻一番，城镇常住居民人均可支配收入增长8.2%，农村常住居民人均可支配收入增长8.5%，居民消费价格涨幅控制在3.5%以内，城镇新增就业岗位2万个，城镇登记失业率控制在4%以内，PM2.5年均浓度比上年降低1微克/立方米，确保完成国家下达的节能减排降碳控制目标。为实现上述目标，要重点抓好以下工作：

（一）争当改革开放的开拓者，在高标准高质量建设自贸试验区和中国特色自由贸易港方面引领示范

1.积极推进制度创新。全面实行外资准入前国民待遇加负面清单管理制度，完善外商投资企业设立商务备案与工商登记“一口办理”，在国际贸易“单一窗口”建设和应用上走在全省前列。深化口岸管理体制改革，推行跨部门一次性联合检查，持续提升通关效率。建立健全以信用监管为核心、与负面清单管理方式相适应的监管体系，强化事中事后监管。加快金融开放创新，保障海南自由贸易账户体系在三亚顺利运行，推进跨境人民币和离岸金融业务发展创新，积极申请开展合格境内投资者境外投资业务，探索设立特色交易场所。依托国家重大科研创新平台，集聚创新要素，鼓励政府机关、市场主体、科研单位和个人大胆开展创新活动，及时总结推广基层创新经验。

2.提升对外开放水平。围绕种业、旅游、医疗、教育、体育、文化、金融等重点领域，深化现代农业、高新技术产业、现代服务业对外开放，加快发展更高层次、更高水平的开放型经济。加快完善招商引资配套政策体系，充分发挥各区自主招商作用，健全第三方招商、产业链招商机制，瞄准世界500强、中国500强、行业领军企业实施精准招商。严格落实招商项目落地责任制，开通招商项目审批许可绿色通道，做好工商登记、土地供应、行政审批、落地运营、人才服务等“一条龙”工作，高效推进项目开工建设和投产运营。推进三亚海上旅游合作开发基地、三亚海上救援综合基地等项目建设，同“一带一路”沿线国家、地区和城市加强文化、教育、旅游、农业、科技等领域的交流合作。主动融入粤港澳大湾区建设，依托泛珠三角区域等合作机制，加强对粤港澳的招商引资、招才引智。

3.深化市场化改革。出台促进民营经济发展的政策文件，清理废除妨碍统一市场和公平竞争的规定与做法，落实好国家更大规模的减税降费政策和个人所得税新税制，制定切实降低企业成本的政策措施，支持民营企业创新转型发展。拓宽企业融资途径，逐步降低融资成本，支持企业挂牌、上市，提高直接融资比例。建立自由贸易港建设投资基金，发挥财政性资金带动作用，扶持新兴产业企业发展，帮助重点企业解忧纾困，激发民间投资活力。加快推进三亚市国有资本运营平台和国有资本投资平台试点改革，组建三亚交通投资集团公司、三亚发展控股有限公司，筹划组建三亚旅游投资企业上市平台公司。创新知识产权管理制度，推进知识产权综合执法。进一步加强社会信用体系建设，严格执行守信激励和失信惩戒制度。

4.强化重点领域风险防范。完善金融监管协调机制，建立信息共享、监测分析、信用体系等风险防控制度和风险应急响应体系。重点加强房地产等行业的重大金融风险识别，建立抵押登记信息公开制度。严厉打击金融犯罪活动，确保不发生系统性、区域性金融风险。严控政府债务风险，积极化解存量债务，确保政府债务风险指标控制在警戒线以内。强化长期支出责任管理，规范政府引进社会资本的模式，严禁新增隐性债务。进一步加强口岸核心能力建设，优化海关监管方式和进出境安全管理，围绕全球动植物种质资源引进中转基地等重点，加强对生物疫病、走私、非法跨境资金转移等风险的防范。

（二）着力构建现代化经济体系，在推动经济高质量发展方面打造标杆

5.积极推动现代服务业集聚发展。积极引进各类金融机构、金融区域总部，发展离岸金融、财富管理、保险服务等高端金融业态。结合房地产业调控，加快金融产业和产品转型，强化金融对实体经济的服务。加快凤凰海岸、月川、东岸、海罗片区土地整理，积极推进总部经济及中央商务启动区规划建设。加快海棠湾保利C+会展中心建设，推动红塘湾会展中心尽快开工，积极引进、培育国际化的会展专业服务机构，打造三亚国际会展品牌。扎实推进三亚凤凰国际机场保税物流中心、三亚佳翔航空货运农产品加工贸易冷链物流园建设，推动跨境电商产业园项目尽快落地，积极发展现代物流业。依托市中医院高标准打造国际化中医药健康旅游示范区，加快推进南岛国家森林康养基地等项目，积极引进国际化的医疗健康专业服务机构，推进三亚国际旅行卫生保健中心建设，发展特色医疗健康产业。精心打造海南岛国际电影节、三亚国际音乐节等重大活动品牌，带动相关文化产业发展，争取与国际相关城市联合举办三亚国际时装节等文化活动。举办好FE国际汽联电动方程式锦标赛等国际赛事，筹备好2020年第六届亚洲沙滩运动会，探索发展竞猜型体育彩票和大型国际赛事即开彩票。加快推进国际体育产业园项目，建设国家体育旅游示范区和国家体育训练南方基地。

6.加快发展高新技术产业。全力推进“一港三城一基地”规划建设，组建崖州湾科技城法定管理机构，创新市场化运营机制和土地开发模式。推动深海技术实验室正式运行，推动中船重工、中船工业等企业重大项目建设，积极引进中国海洋大学等国内外知名高校和科研机构，打造国家深海基地南方中心和国际一流的深海科技创新平台。加快建设国家南繁科研育种基地、国家热带农业科学中心、全球动植物种质资源引进中转基地，引进和培育一批有影响力的科研机构、种业集团和南繁服务外包企业，推动南繁育种由事业向产业转变，加快实现集南繁种业研发、种业贸易、种业会展、种业科普于一体的全产业链发展。启动海南信息安全基地二期项目建设，集聚网络安全力量，打造国家网络安全南方基地。积极支持三亚遥感信息产业园建设。做大做强互联网信息产业园，深入开展与腾讯、京东等行业领军企业的合作，抓紧推动三亚智谷小镇、腾讯“城市超级大脑”等项目落地，建设“数字三亚”。充分利用海南享受服务外包示范城市政策的机遇，加快建设特色服务出口基地。

7.做强做优热带特色高效农业。结合南繁科技优势和种业贸易发展，围绕热带水果、热带花卉、冬季瓜菜等特色产品，引进和选育优质种源，加快农业供给侧结构性改革，打造国家热带现代农业基地。加强农产品品牌培育、营销和保护，充分发挥“三亚芒果”等国家地理标志产品的优势，加快培育壮大“三亚甜瓜”“三亚莲雾”“三亚豇豆”等品牌，扩大品牌影响力和市场占有率。加快农产品质量可追溯信息平台建设，推行农业标准化生产和全程质量控制。提升农产品加工规模和深度，拉长农业产业链，提高农产品价值。积极培育扶持农业龙头企业、农民专业合作社等新型农业经营主体，引进具有国际竞争力的现代农业企业。进一步拓展农产品销售网络，加强产销数据对接，推进农产品市场建设，鼓励农村电商平台创新发展。进一步完善农业金融保险服务。

8.高质量发展海洋经济。开展海洋经济普查，摸清海洋资源家底，推动海洋和深海产业集群化发展，让海洋资源优势转化为经济优势。推动渔业捕捞向深远海发展，着力发展深水网箱、深海智能化养殖，启动水产种苗养殖产业园规划建设，提升海产品储存能力和加工水平，打造三亚海产品品牌。加快推进天涯海角、西岛和崖州湾科研示范基地等现代化海洋牧场项目，发挥其生态修复、休闲观光功能，推动休闲渔业发展。加强海洋科技特别是深海科技研究，推进“智慧海洋”建设，提升海洋资源开发和服务保障能力，加快培育海洋生物、海水淡化与综合利用、海洋能源、海洋工程装备研发与应用等新兴产业，发展邮轮、游艇等船舶维修业务。

（三）加快旅游消费提质升级，在建设国际旅游消费中心方面创新先行

9.培育丰富多彩的旅游消费新业态。继续推动鹿回头等传统景区改造提升，推动天涯海角、西岛旅游区、热带天堂森林公园创建5A级景区，推进海棠湾“国家海岸”开发建设，高标准规划改造亚龙湾国际旅游度假区。推进HelloKitty主题乐园等项目落地，争取今年新引入3～5家中外合资旅行社。加快国际邮轮母港建设，积极开展邮轮公海游试点，拓展三亚国际旅游“一程多站”航线，开发环海南岛邮轮航线和面向太平洋、印度洋的跨国邮轮旅游航线。成立邮轮旅游联盟，打造21世纪海上丝绸之路邮轮旅游交流平台。推进琼港澳游艇自由行，按照“定点停靠、就近联检”的口岸管理模式，简化游艇出入境手续。结合游艇租赁和旅游，积极推进海上婚庆等新业态发展。争取进一步放活免税购物政策，积极引进国际品牌免税店。着力引进全世界最优质的产品，同时加强特色地域产品开发和品牌打造，吸引全世界游客前来消费。全面深化旅游与健康、文化、会展、体育等产业的融合发展，着力打造以观光旅游为基础、休闲度假为重点、文体旅游、会展旅游和健康旅游为特色的旅游产业体系。

10.创建国际一流的旅游消费环境。按照国际通行标准，加快旅游设施和要素改造，推动旅游服务与国际接轨，推进绕城高速公路、国省干道和重点湾区酒店群多语种旅游标识标牌建设，在旅游行业开展多种外语培训，配备多语种翻译设备。加快完善全市商业网点体系，高标准建设具有国际影响力的高品位步行街、文化娱乐场所、大型消费商圈，优化区级消费商圈布局，完善“互联网+”消费生态体系，推进智能店铺和智慧商圈建设。加强旅游消费市场监管，继续优化“科技治旅”技术手段和工作机制，进一步提升监管效率。提升三亚“诚信商家”体系品牌的认知度，打造三亚放心游诚信品牌。

11.进一步拓展旅游国际市场。深化三亚空域精细化管理改革，优化空域结构，推进凤凰机场进离场航路分离，力争实现机场航班容量扩容。加强第五航权航线研究布局，今年争取新开境外航线15条以上。用好59国入境免签政策，结合航线开辟，与国际大旅行商和主流媒体建立战略合作关系，针对不同的目标客源地制定专属营销方案，争取今年全市入境游客增长20%以上。

（四）以“多规合一”引领城乡建设，在乡村振兴与区域协调发展方面争做表率

12.切实加强规划管控。完善“多规合一”动态维护，推动“多规合一”成果运用。启动新一轮三亚城市总体规划修编工作，加快完成《三亚市总体城市设计（2018-2030）》。完成第三次国土资源调查工作，编制三亚市国土空间规划，优化国土空间资源配置，严格开发边界、生态保护红线和永久基本农田红线管控。大力强化城市规划建设管理，严格落实城市设计和建筑风貌管理实施细则的要求。完善村庄规划编制和管控，做到农房逢建必报。保持打击违法用地、违法建设高压态势，深入开展“两违”整治，确保今年“两违”零增长。

13.加快构建现代基础设施体系。成立三亚新机场工作专班，加快推进新机场选址等前期工作。推动崖州中心渔港定位调整与建设升级，着力完善各类配套设施，打造我省南部渔货交易中心和渔港经济区。完善凤凰岛国际邮轮母港配套设施与功能布局。加强南山港配套设施建设，积极争取南山港设立海关监管场所并申请对外开放。实施解放一路等市政道路改造，加快推进胜利路改造工程，确保年底前实现三横路功能性通车，完善城市路网。大力推进自然村通硬化路、窄路面拓宽、安全防护、危桥改造和旅游公路建设等工作，加快自然村“村村通公交”工程建设。加快建设第二气源站，推进崖州、育才、高峰、回新等11个片区天然气管道建设，促进燃气管网由主城区向农村延伸。加快完成青田水厂原水管道扩建工程，大力推进西水中调一期工程建设，增强后备供水能力。加快建设北部山区饮水安全工程配套管网，实施农村供水巩固提升工程，尽早实现农村供水“同网同质同价同服务”全覆盖。加快完善农田水利“毛细血管”，改善灌溉、排涝末端不畅的问题。积极开展智能电网建设，确保用电安全，争取做到中心城区全年户均停电时间不超过1小时。加快实施信息进村入户工程，推动城乡高速光纤和4G信号实现全覆盖，开展5G建设试点。

14.大力实施乡村振兴战略。深入开展农村人居环境整治，积极推进污水垃圾收集处理、厕所革命、村容村貌提升等工作。以产业为核心，探索建立“一村一企”帮扶模式，加快推进全市92个行政村、491个自然村美丽乡村建设。按照全域旅游的要求，挖掘特色资源，引入多元市场主体，优化利益联结机制，因地制宜打造特色鲜明的乡村游产品，把亚龙湾玫瑰风情小镇和中廖村、博后村、西岛渔村等打造成全省标杆性项目。深化农村土地征收、集体经营性建设用地入市和宅基地制度改革，解决好集体经济预留用地问题，全面铺开农村集体产权制度改革。支持利用闲置农房发展民宿项目，打造特色田园综合体，大力发展共享农庄。

15.加快“大三亚”经济圈建设。配合推进“大三亚”经济圈一体化规划编制，加快区域基础设施、产业发展、生态环保、公共服务一体化进程。全力保障海南省环岛旅游公路陵水土福湾至三亚海棠湾通道、老西线铁路三亚至乐东段公交化旅游化改造等重点项目建设，谋划三亚第二绕城高速公路，打造“大三亚”水上交通体系。深化跨区域旅游治理管理，开展联合宣传推介和联合市场监管。加强南繁和农业领域协调合作，开展好跨市县横向生态保护补偿试点工作。协调推进“大三亚”公共服务资源共建共享。

（五）精心打造生态宜居城市，在推进人与自然和谐发展方面不断探索

16.扎实推进中央环保督察和国家海洋督察反馈意见整改。坚决落实中央环保督察93项整改措施、国家海洋督察38项整改措施，对照落实情况台账，实行销号备案管理。加大督查考核力度，对已完成的信访交办案件和反馈意见整改任务进行“回头看”，确保真整改，不反弹。组织开展全市环境突出问题排查整治，确保新的问题早发现、早解决。

17.坚决打好污染防治攻坚战。严格落实大气污染防治“六个严禁、两个推进”的要求，巩固提升大气环境质量。年内完成荔枝沟水质净化二厂建设，开工建设育才水质净化厂、三亚市人才城水质净化厂，加快完善全市污水主管网和配套支管网。继续推进全市农村生活污水处理设施建设，确保今年内农村生活污水处理设施覆盖率达到90%。完成高园水库—大茅水水系连通工程，开工建设亚龙溪综合治理、三亚河口清淤疏浚工程，推进小型水库及山塘除险加固。继续推进11条城镇内河水环境治理，持续提升水环境质量。加强海岸带、海湾生态保护和修复，实施近岸海域综合治理，抓好崖州中心渔港生态环境整治。加快推进三亚河国家湿地公园建设，加强三亚河、铁炉港红树林保护区的建设、修复和管理。积极推进凤凰岭、福岭、北山、凤岭森林公园建设，继续实施绿化宝岛和废弃矿坑生态修复工程，开展侵占公益林种植经济林清退工作，严禁开山毁林等破坏行为。建立农业面源污染防治长效机制，加强畜禽养殖污染防治，积极创建国家农业绿色发展试点先行区，争创国家生态循环农业示范市。

18.建立健全生态文明制度体系。编制生态保护红线、环境质量底线、资源利用上线、生态环境准入清单“三线一单”，严格落实相关管控措施。完善环境监测网络，建立资源环境承载能力监测预警机制。实行生态文明建设和环境保护目标责任制，健全生态文明考核评价体系，出台党政领导干部生态环境损害责任追究实施细则。建立环境污染“黑名单”、环保信用评价、信息强制披露、严查重罚等制度。

19.推动形成绿色生产生活方式。建立健全产业准入负面清单，全面禁止高能耗、高污染、高排放产业和低端制造业发展，构建绿色产业体系。严格执行项目单位土地面积投资强度、产出强度等标准，提高资源节约集约利用水平。实施能源消费总量和强度双控行动。加快建设国家第三批低碳试点城市，大力推进三亚蜈支洲岛、海南中学三亚学校、三亚低碳智慧能源综合利用海棠湾示范区等零碳低碳示范区项目试点。大力推广装配式建筑，推行高星级的绿色建筑。大力推行绿色低碳交通，提前实施国家第六阶段机动车排放标准，加快完善充电基础设施网络，推广新能源汽车和节能环保汽车。加快推进生活垃圾焚烧发电厂扩建工程及配套项目、渗滤液处理站增容工程、粪便处理厂等环保设施建设，推进三亚循环经济产业园建设，加快构建覆盖生活垃圾、餐厨垃圾、建筑垃圾、医疗废弃物等各类垃圾的处理体系。继续开展生活垃圾分类试点。严格落实禁塑措施。

（六）坚持以人民为中心的发展思想，在保障和改善民生方面全力以赴

20.坚决打赢精准脱贫攻坚战。以《三亚市打赢脱贫攻坚战三年行动计划》为统领，实施产业、旅游、就业、生态、教育、危房改造、综合保障、扶智扶志、社会扶贫等十大扶贫工程，在全市范围内消除绝对贫困，对因病因残致贫群众做好兜底保障。注重扶志、扶智相结合，突出抓好产业扶贫、就业扶贫，贫困户产业覆盖率要保持100%，要确保有劳动能力且有就业意愿的贫困家庭至少1人就业。继续完善和落实好“两不愁三保障”等各项扶贫政策体系，抓好农村最低生活保障制度与扶贫开发政策有效衔接。扎实推进已脱贫人口的分类巩固，重点研究解决收入水平略高于建档立卡贫困户的群体缺乏政策支持的问题。

21.优先发展教育事业。新建17所幼儿园、3所小学和3所中学。确保人大附中三亚学校、三亚寰岛实验小学秋季开学，加快推进上海外国语大学三亚附中项目建设，积极引进一批优质国际教育合作交流项目。开展“好校长、好教师”选拔培训，建立教育竞争激励机制。着力增强农村地区师资力量，提升边远地区教师待遇。积极支持海南热带海洋学院、三亚学院等本地高校建设发展，培育打造特色学科，提升职业教育水平，大力培养适应发展新形势的新人才。

22.提升医疗卫生服务水平。加快市中医院改扩建一期工程、省第三人民医院门诊综合楼和北大口腔医院三亚分院建设，推动市妇幼保健院与上海儿童医学中心深度合作，打造一批国家级、省级重点专科。完成全市83个基层医疗卫生机构标准化建设，继续推进紧密型医联体建设，加强基层医疗队伍建设，落实分级诊疗，构建15分钟城市健康服务圈、30分钟乡村健康服务圈。积极推进健康城市创建工作。

23.完善公共文化服务。大力推进市文化中心、体育中心、新闻媒体中心和三亚歌剧院建设，加快区级文化馆、图书馆、全民健身中心规划建设，积极开展群众性文化体育活动，积极创建国家第四批公共文化服务体系示范区和“东亚文化之都”。挖掘和传承以崖州文化为代表的历史文化，重点推进崖州古城建设。创作现代文艺精品，推动文化大繁荣大发展，丰富市民游客的精神生活。

24.织密扎牢民生保障网。突出重点稳就业，加快建设公共就业创业服务平台，重点做好高校毕业生、农民工、退役军人、就业困难人员等群体就业工作。对接市场和企业用工需求，开展“订单培训”“定向培训”，推行终身职业技能培训制度。用心用情抓社保，有序推进社保制度改革，全面实施“全民参保登记计划”，进一步扩大社保覆盖面，解决好引进人才社保转移接续问题。加快发展企业年金、职业年金等，完善多层次社保体系。认真落实国家有关政策，把更多救命救急的好药纳入医保。合理布局康复养老机构，大力发展社区健康养老服务、居家养老服务，鼓励民办养老机构发展。加快抱坡新城等保障性住房项目建设，抓好房源分配和后续管理。继续推进南边海环河口、凤凰海岸、西瓜村、芒果村等片区棚改旧改，加快棚改安置区建设进度，改造200户农村危房。继续做好“菜篮子”工作，积极推进政府应急调控基地建设，启动岛内外“飞地种植蔬菜”工作，进一步完善平价网点布局，丰富产品品种，保证产品质量，稳定保供稳价体系，全力做好台风、暴雨等异常天气和节庆假日等特殊时段的保供稳价工作。

25.加强和创新社会治理。深入开展社会文明大行动和全国文明城市创建工作，推进“五大文明创建”，抓好“九个专项行动”，着力提升市民文明素质和社会文明水平。加快推进道路机非分离，因地制宜建设停车场、施划停车位，规范交通秩序。积极开展“三无”小区综合整治和城区危旧房改造。继续推进综治中心、雪亮工程、网格化管理“三位一体”新机制建设，深化“路长制”“警保联控”等警务改革创新，不断完善社会治安防控体系，加强社会面防控。充分发挥互联网在社会治理中的重要作用，加快形成集信息共享、部门联动、综合研判、跟踪督办、应急处置于一体的工作体系。扎实推进扫黑除恶专项斗争，严防发生暴恐事件。坚决打赢禁毒三年大会战收官之战。严厉打击“菜霸”“砂霸”“运霸”等违法行为，营造安全有序的市场环境。进一步强化安全生产责任制，加强重点行业领域安全隐患排查和专项整治，严防重特大安全事故。做好信访工作，深入开展矛盾纠纷大排查大化解活动。推进军民融合深度发展，在产业融合、公共服务融合、基础设施共建共享等方面迈出新步伐，做好双拥共建、国防教育、国防动员、人民防空、民兵预备役等工作，守好祖国南大门。进一步强化食药安全、民族宗教、外事侨务、对台、档案、气象、防震减灾等工作，支持工青妇和老龄等事业取得新进步。

（七）全面加强政府自身建设，在打造一流营商环境方面奋勇争先

26.加强政治建设和廉政建设。坚持用习近平新时代中国特色社会主义思想武装头脑，增强“四个意识”，坚定“四个自信”，做到“两个维护”，坚决贯彻落实中央方针政策以及省委、省政府和市委的决策部署。坚持抓好作风建设，把纪律和规矩挺在前面，严格落实“一岗双责”，切实履行全面从严治党主体责任。尽心尽力为企业服务，干干净净与企业交往，努力实现“亲”的目标，坚决守好“清”的底线。深化标本兼治，坚决惩治腐败，自觉接受市委、市纪委监委和广大人民群众的监督，坚决查处各类违法违纪案件，坚决纠正损害群众利益的不正之风。

27.提升政府执行力。认真贯彻省政府提高政府执行力十五条措施的精神，狠抓执行力建设。深化机构改革，理顺行政职能，科学配置行政资源。结合自贸试验区建设的新形势、新需要，积极开展优秀干部招录、延揽，全面加强现有人员培训、锻炼，提升干部队伍的综合素质和执行能力。进一步推进强区扩权改革，理顺市区两级职责权限，提升权限下放的“含金量”，推动权责匹配，加强基层力量配备和业务培训指导，增强基层执行能力。加强第三方考核评估、动态考核和考核结果运用，做到奖优罚劣。

28.着力打造法治化营商环境。按照《三亚市法治政府建设实施方案》的部署，大力建设法治政府。积极配合市人大开展立法工作，加强和改进政府规章的制定工作。坚持依法决策、民主决策、科学决策，特别是在重大决策时，要严格执行公众参与、专家论证、风险评估、合法性审查和集体讨论决定等法定程序，保障决策符合实际，提升政策的可操作性和连续性、稳定性。加强政府诚信建设，努力解决“新官不理旧账”等问题，依法依规妥善处理历史遗留问题，保障企业合法权益。自觉接受人大、政协监督，提高人大代表建议和政协提案的办理质量。认真执行市人大及其常委会的决议、决定和审议意见，并按照程序及时向人大常委会报告执行情况。加强同民主党派、工商联、无党派人士、人民团体和各界人士的联系，广泛听取意见建议。支持开展司法监督，充分发挥审计监督和社会监督作用。

29.着力打造国际化营商环境。积极推动投资贸易监管与国际接轨，引导经济活动遵循国际规则和惯例。大力引进国际化的法律、仲裁、会计、审计、知识产权、评级服务、检测认证以及规划、建筑设计、人力资源服务等专业服务机构，构建多元化的国际商事纠纷解决机制。认真落实“百万人才进海南”行动计划，实行更加积极、开放的人才政策，争取探索开展国际人才管理改革试点，服务好港澳台及外籍人才就业创业。加强本地人才的培养，用好用活“候鸟型”人才。大力实施人才安居工程，围绕教育、医疗、文化等重点，加快构建适应国内外人才需求的公共服务体系。

30.着力打造便利化营商环境。结合国家营商环境评价工作，进一步推进“放管服”改革，严格执行《三亚市优化审批服务20条》，提升审批服务效能。在“多规合一”框架下，优化建设项目审批，推行“极简审批”，实施“两验终验”。继续完善市、区、村三级政务服务体系，在全市政务服务事项“十个统一”的基础上，开展事项标准化“颗粒度”细化梳理工作。依托12345行政审批专席和三亚市政务服务“一张审批网”，进一步推进市、区“二级融合、全市通办”。提升市区两级政务服务大厅“一站式”功能，年底前政务服务事项进驻综合性实体政务大厅基本实现“应进必进”，70%以上的政务服务事项实现“一窗受理”。加强政务服务便利化大数据治理体系建设，打通部门信息壁垒，推进系统对接和信息共享，持续完善政务服务前端应用体系。

各位代表！新时代赋予新使命，新征程要有新作为。建设自由贸易试验区和中国特色自由贸易港的历史责任就落在我们这代人的肩上。让我们更加紧密地团结在以习近平同志为核心的党中央周围，在省委、省政府和中共三亚市委的领导下，紧紧依靠全市人民，以高度的责任感和使命感，坚定信心，真抓实干，锐意进取，努力把三亚打造成为新时代全面深化改革开放的新标杆，向党中央、省委省政府和全市人民交上一份满意的答卷！
